# Supplementary material for: Somatic Embryogenesis Initiation in Sugi (Japanese Cedar, Cryptomeria japonica D. Don): Responses from Male-Fertile, Male-Sterile, and Polycross-Pollinated-Derived Seed Explants
Source: Plants (Basel). 2021 Feb 19;10(2):398. doi: 10.3390/plants10020398 (PMC7922571; doi:10.3390/plants10020398)
Supplement: Supplementary file 1 [file plants-10-00398-s001.pdf]

plants

MDPI

Article

Somatic Embryogenesis Initiation in Sugi (Japanese Cedar, *Cryptomeria japonica* D. Don): Responses from Male-Fertile, Male-Sterile, and Polycross-Pollinated-Derived Seed Explants Supplementary Materials

Supplementary Table S1: Somatic embryogenesis (SE) initiation frequency from open-pollinated-derived seeds of male-fertile families of sugi. The data represent the explants with SE initiation response and the total number of explants tested, and the numbers in the parentheses represent the initiation frequency (%) for each mother tree by seed collection year.

Mother Tree	SE Initiation Frequency (%) by Seed Collection Year									
	1997	1998	1999	2002	2003	2004	2005	2006	2008	2011
"Chiyoda 327"	12/100 (12.00)									
"Kuji 6"						9/60 (15.00)				
"Kuji 9"				1/74 (1.35)						
"Kuji 14"					161/320 (50.31)	75/240 (31.25)	21/48 (43.75)	80/180 (44.44)	72/196 (36.73)	136/240 (56.67)
"Kuji 17"								11/80 (13.75)	6/184 (3.26)	
"Kuji 39"				5/94 (5.32)						
"Naka 3"					35/144 (24.31)		6/48 (12.50)			
"Naka 5"				1/74 (1.35)				9/48 (18.75)		
"Naka 6"					18/78 (23.08)					
"Nihari 2"				20/228 (8.77)	20/78 (25.64)	9/72 (12.50)	22/48 (45.83)			
"Taga 2"				12/202 (5.94)			170/252 (67.46)	9/48 (18.75)		
"Taga 4"						28/144 (19.44)	9/48 (18.75)			
"Taga 10"					2/24 (8.33)					
"Taga 14"								79/220 (35.91)		116/360 (32.22)
"Tsukuba 2"						13/144 (9.03)	14/48 (29.17)			
"Yamazaki 5"	23/156 (14.74)									
"Yanase 104"	20/150 (13.33)	37/288 (12.85)	48/312 (15.38)							
Total	55/406 (13.55)	37/288 (12.85)	48/312 (15.38)	39/672 (5.80)	236/644 (36.65)	134/660 (20.30)	242/492 (49.19)	188/576 (32.64)	78/380 (20.25)	252/600 (42.00)

Supplementary Table S2: Somatic embryogenesis (SE) initiation frequency from sugi seed families carrying the male sterility gene *MS1* or *MS2*. The data represent the explants with SE initiation response and the total number of explants

tested, and the numbers in the parentheses represent the initiation frequency (%) for each seed family by seed collection year.

**Seed Family**	**Male Sterility Gene**	**SE Initiation Frequency (%) by Seed Collection Year**		
		**2016**	**2017**	**2018**
♀ **"Shindai 3"**<br>♂ **"Suzu 2"**	MS1	212/1,236)<br>(17.15)	284/707<br>(40.17)	
♀ "Fukushima-funen 1"<br>♂ "S3-37(1)"	MS1		81/1,056<br>(7.67)	
♀ "Fukushima-funen 1"<br>♂ "Oi 7"	MS1		355/936<br>(37.93)	
♀ "Fukushima-funen 1"<br>♂ "S3-118(2)"	MS1		416/1,017<br>(40.90)	
♀ "S1S1-35"<br>♂ "Gosenshi 1"	MS2			10/74<br>(13.51)
♀ "S1S1-23(1)"<br>♂ "Gosenshi 1"	MS2			1/36<br>(2.78)
♀ "S1S1-10(1)"<br>♂ "Gosenshi 1"	MS2			54/186<br>(29.03)
♀ "S1S1-51(1)"<br>♂ "Gosenshi 1"	MS2			14/347<br>(4.03)
Total		212/1,236)<br>(17.15)	1,136/3,716<br>(30.57)	79/643<br>(12.29)

Supplementary Tables S3: Mother trees and pollen parents used for polycross-pollinated-derived seeds of sugi.

Polycross Family	Collection Year	Mother Tree	Pollen Parent
"S 11" × "3 Mix"	2019	"Shindai 11"	"Higashikanbara 5"
			"Iwafune 9"
			"Nakakubiki 2"
"S 11" × "10 Mix"	2019	"Shindai 11"	"Higashikanbara 5"
			"Iwafune 17"
			"Minamikanbara 3"
			"Iwafune 9"
			"Ryotsushi 1"
			"Iwafune 8"
			"Nakakubiki 2"
			"Iwafune 16"
			"Iwafune 2"
			"Tokamatishi 1"
"S 1" × "3 Mix"	2020	"Shindai 1"	"Iwafune 8"
			"Ryotsushi 1"
			"Nakakubiki 2"
"S 1" × "9 Mix"	2020	"Shindai 1"	"Iwafune 2"
			"Iwafune 8"
			"Iwafune 12"
			"Iwafune 16"
			"Iwafune 17"
			"Ryotsushi 1"
			"Nakakubiki 2"
			"Iwafune (Kan) 1"
			"Nakakubiki 4"
"G 1" × "10 Mix"	2020	"Gosenshi 1"	"Iwafune 2"
			"Iwafune 5"
			"Iwafune 8"
			"Iwafune 12"
			"Iwafune 16"
			"Iwafune 17"
			"Ryotsushi 1"
			"Nakakubiki 2"
			"Iwafune (Kan) 1"
			"Nakakubiki 4"

Supplementary Table S4: Somatic embryogenesis (SE) initiation responses from polycross-pollinated-derived seed families of sugi. The data represent the explants with SE initiation response and the total number of explants tested, and the numbers in the parentheses represent the initiation frequency (%) for each polycross family by seed collection year.

Polycross Family [1]	SE Initiation Frequency (%) by Seed Collection Year		
	**2019** [2]	**2019** [3]	**2020**
"S 1" × "3 Mix"			32/286 (11.19)
"S 1" × "9 Mix"			28/268 (10.45)
"S 11" × "3 Mix"	90/216 (41.67)	1,211/2,064 (58.67)	
"S 11" × "10 Mix"	20/55 (36.37)	1,011/2,013 (50.22)	
"G 1" × "10 Mix"			40/478 (8.37%)
Total	110/271 (40.59)	2,222/4,077 (54.50)	100/1,032 (9.69)

[1] Mother trees and pollen parents used for polycross seed families are shown in Supplementary Table S3; [2] results of the Niigata Laboratory; [3] results of the Tsukuba Laboratory.
